# The Relationship Between Outdoor Activity and Health in Older Adults Using GPS

**DOI:** 10.3390/ijerph9124615

**Published:** 2012-12-11

**Authors:** Jacqueline Kerr, Simon Marshall, Suneeta Godbole, Suvi Neukam, Katie Crist, Kari Wasilenko, Shahrokh Golshan, David Buchner

**Affiliations:** 1 Department of Family and Preventive Medicine, UCSD, 9500 Gilman Drive, La Jolla, CA 92093, USA; E-Mails: sjmarshall@ucsd.edu (S.M); sgodbole@ucsd.edu (S.G.); kcrist@ucsd.edu (K.C.); kwasilenko@ucsd.edu (K.W.); 2 University of New England College of Osteopathic Medicine, 11 Hills Beach Rd, Biddeford, ME 04005, USA; E-Mail: sneukam@une.edu; 3 Department of Psychiatry, UCSD, 9500 Gilman Drive La Jolla, CA 92093, USA; E-Mail: sgolshan@ucsd.edu; 4 Department of Kinesiology and Community Health, University of Illinois at Urbana Champaign, Champaign, IL 61820, USA; E-Mail: dbuchner@illinois.edu

**Keywords:** physical activity, older adults, outdoor time, accelerometry, Global Positioning System (GPS), physical functioning, cognitive functioning, health

## Abstract

Physical activity (PA) provides health benefits in older adults. Research suggests that exposure to nature and time spent outdoors may also have effects on health. Older adults are the least active segment of our population, and are likely to spend less time outdoors than other age groups. The relationship between time spent in PA, outdoor time, and various health outcomes was assessed for 117 older adults living in retirement communities. Participants wore an accelerometer and GPS device for 7 days. They also completed assessments of physical, cognitive, and emotional functioning. Analyses of variance were employed with a main and interaction effect tested for ±30 min PA and outdoor time. Significant differences were found for those who spent >30 min in PA or outdoors for depressive symptoms, fear of falling, and self-reported functioning. Time to complete a 400 m walk was significantly different by PA time only. QoL and cognitive functioning scores were not significantly different. The interactions were also not significant. This study is one of the first to demonstrate the feasibility of using accelerometer and GPS data concurrently to assess PA location in older adults. Future analyses will shed light on potential causal relationships and could inform guidelines for outdoor activity.

## 1. Introduction

Regular physical activity (PA) provides extensive health benefits in adults, including reduced risk of depressive illness, cognitive impairment, and functional limitations [1]. Hence, both the USA and World Health Organization have recently issued public health guidelines for PA [2,3]. Walking for 30 min or more a day is widely recommended as a way to meet the PA guidelines.

A growing body of research suggests that exposure to nature and time outdoors also provides health benefits, particularly for mental health and an improved sense of well-being [4,5,6]. A popular explanation is biophilia—the idea that humans have an innate connection and attraction to Nature [7]. However, there are not yet public health guidelines that recommend spending a certain amount of time outdoors or in natural environments. Additionally, the mechanisms by which exposure to nature may improve health are not well studied. 

If PA and outdoor time both have independent effects on health public health guidelines should encourage adults to perform PA outdoors as a more efficient way to obtain both sets of health benefits. It is currently unknown if this advice is justified. Since the mechanism relating outdoor time to health is unknown, it is not clear whether the benefits of PA and outdoor time are additive or multiplicative. 

The purposes of the current study are: (1) to assess whether 30+ min of outdoor time per day is associated with health benefits in older adults; and (2) to determine if health benefits of outdoor time are additive to benefits of 30+ min/day of PA. The study used an objective measure of PA (accelerometry) and an objective measure of outdoor time (GPS); previous studies have relied on self-report [6]. The study focused on older adults, as the issue of promoting outdoor activity appears most important in this age group with older adults having the lowest levels of PA of any age group, and likely to spend less time outdoors than other age groups [8,9]. If benefits of outdoor PA can be shown for older adults, this specific activity could be included in population and practice guidelines and efforts to provide safe outdoor environment for older adults would be required.

## 2. Methods

### 2.1. Participants

Participants were older adults living in five continuing care retirement communities (CCRC) in San Diego County who volunteered for a randomized control trial. They were randomized to a PA program or a successful aging program for a 6 month intervention. This is an ongoing study and only baseline data from both groups are presented here. Study participants were CCRC residents aged 65 years and above. Eligibility criteria included: ability to speak and read English; ability to complete written assessments; ability to provide informed consent; no history of falls within the past 12 months that resulted in hospitalization; ability to walk 20 m without human assistance, completion of the Timed Up & Go Test in less than 30 s; ability to read survey questions; and completion of a post-consent comprehension test.

### 2.2. Measures and Procedures

Participants were asked to wear a GPS and accelerometer for 7 days during waking hours. PA was measured objectively with the Actigraph accelerometer (MTI, Inc.) because it is very small, we have extensive experience with its use, and validation and calibration data are available [10,11]. We used the Actigraph 3X-plus model collecting data at 30 Hertz in three planes of movement. Participants were required to wear the accelerometer for a minimum of 10 h a day [12]. Participants were asked to re-wear the device if the criteria were not met on at least 4 days. The data were processed with the Actigraph Actilife 5 software and aggregated to 60 s. We used 90 consecutive zeros with a 2 min threshold to screen for non-wear time [13]. We applied valid PA intensity thresholds that operate at the minute level. A cut off of 1,040 counts per minute was employed to assess PA that occurred at moderate and high-light intensity [14,15]. The accelerometers were initialized on a computer synched to the Universal Time Clock time so that the time stamps on the accelerometer and GPS could be matched.

Participants simultaneously wore a Qstarz BT1000X GPS device. A GPS device logs *X,Y* location coordinates, distance, speed, elevation, and time. The Qstarz has an accuracy of 3 m. We configured the device to record additional satellite information (Signal to Noise Ratio: SNR) which enables us to detect outdoor locations. The less noise, the more likely the participant is outdoors. The device recorded every 15 s. The Qstarz GPS is smaller than a cell phone and is worn inside a pouch on the same belt as the accelerometer at all times. Participants charged this device every evening, according to protocols developed by our staff to maximize compliance [16]. 

Accelerometer and GPS data were merged by their time stamps using the Personal Activity Location Measurement System (PALMS) software, and filtered for spurious data points using standard algorithms [16]. Outdoor time was calculated based on the ratio of GPS-detected satellites to the total number of orbiting satellites. The algorithms have been validated in two studies [17,18]. A threshold of 250 was employed for the SNR values. Values below 250 indicate increased interference usually from signal blockage by building materials which is likely to represent being indoors. Sensitivity and specificity is over 80% for this value [17,18]. Time spent in vehicle (determined by GPS speed and accelerometer counts below 200) was excluded. Total time spent outdoors and time spent in PA across the wear days were divided by the number of valid days worn to provide a minutes per day value. 

#### 2.2.1. Emotional Functioning

For aging adults with chronic illness, the quality of their remaining years may be more important than the time they have left [19]. Quality of life was measured with the Perceived Quality of Life Scale (PQOL). An 11-item version of the PQOL was examined for reliability and validity [20]. The measure was developed using formative research with older adults and persons with disabilities. Items included are satisfaction with: physical health, caring for yourself, thinking and remembering, walking, getting outside, carrying on conversation, seeing and talking to friends, helping family and friends, contributing to the community, recreation and leisure time, respect from others, and meaning and purpose in life. Items were scored on a 1 (extremely unhappy) to 5 (extremely happy) scale. The mean score across all items were used for analysis.

PA has been associated with lowered risk of depression among older adults [21,22,23]. Depression was measured with CESD short form [24]. Participants respond to questions on a 0–3 point scale based on how they have felt or behaved over the past week. Items include: I was bothered by things that usually don’t bother me; I had trouble keeping my mind on what I was doing; I felt depressed; I felt that everything I did was an effort; I felt hopeful about the future; I felt fearful; My sleep was restless; I was happy; I felt lonely; I could not get “going”. Summed scores 0–30 were used for analysis. Scores 10 or above are considered representative of depressive symptomology, higher scores indicating greater symptoms.

*Cognitive functioning* [25,26,27,28]: was assessed with the Trail Making Test (TMT) Part A and B, commonly used in older adults to assess cognitive function and as an indicator of functional differences. Participants completed paths from ascending numbers (Part A) and alternating number to letter (Part B). TMT-B is specifically related to executive functioning; however, spatial organization, psychomotor speed, and visual scanning are also involved. TMT scores have been related to PA in older adults [29,30,31]. We focused on the time to complete TMT B in the current analyses.

Fear of falling has been related to PA and time spent outdoors in older adults [32]. The 16-item Falls Efficacy Scale-International (FES-I) has been shown to have excellent reliability and construct validity [33]. Participants rate their concern for falling (1 = Not at all concerned; 5 = Very concerned) when performing the following tasks: cleaning the house; getting dressed or undressed; preparing simple meals; taking a bath or shower, going to the shop; getting in or out of a chair, going up or down stairs, walking around the neighborhood; reaching for something above your head or on the ground; going to answer the telephone before it stops ringing; walking on a slippery surface; visiting a friend or relative; walking in a place with crowds; walking on an uneven surface; walking up or down a slope; going out to a social event. Sum scores were analyzed; scores greater than 23 indicate higher concern of falling.

#### 2.2.2. Physical Functioning

Cardiorespiratory fitness was measured with the 400 Meter Walk Test (400MWT) [34]. The test measures time to walk 400 m quickly, without running; participants can rest if needed. Tests were performed on a marked course in each community. 

The *Late Life Functioning Disability Instrument* (LLFDI) [35] was used to determine self-reported functional performance and physical disability. The survey asked participants to rate their difficulty in performing various tasks (walking a mile, getting in and out of a car, *etc.*). The self-reported responses were scored with 5 = no difficulty, 4 = a little difficulty, 3 = some difficulty, 2 = quite a lot of difficulty, 1 = cannot do. The sum of 9 questions gave a score range of 9 to 45 (9 = low functional performance and 45 = high functional performance). The sum of all scores was employed in these analyses.

Participants provided demographic characteristics including gender and age.

### 2.3. Analyses

Minutes per day spent in PA and outdoors were both highly skewed therefore a dichotomous variable was created for each of the independent variables: time in PA and time outdoors. We employed a 30 min cut off for each behavior. The PA guidelines suggest 30 min of activity per day for health benefits [23]. Studies show that even short periods of time in sunlight can improve Vitamin D levels [36]. In order to control for exposure time, we therefore also selected 30 min for outdoor time which provided an equal unit of measurement.

Analyses of variance were employed with PA and outdoor time tested as main independent effects. The interaction effect between PA and outdoor time was also explored. The main effect tested whether PA or outdoor time were related to the continuous health outcomes, when both were entered into the model *i.e.*, independent of each other. The interaction tested whether participants with both PA and outdoor time were different from those with PA or outdoor time or different from those with neither. Gender and age were entered as covariates. Models were tested for each continuous dependent outcome: depression, QoL, 400MWT, fear of falls and TMT-B. Means for the 2 by 2 design of outdoors time (less than 30 min and 30+ min) and PA times (less than 30 min and 30+ min) are presented in the figure.

## 3. Results

A total of 117 older adults completed the data collection procedures out of 125 participants (94%) enrolled in the study at the time of analysis. Participants did not complete measures due to illness or were excluded if they did not meet the wear time criteria above A total of 69% were female, the average age was 83.3 (SD 6.8). The majority were high income Caucasians. Table 1 presents the descriptive statistics for the two groups tested as the main effects, ±30 mins PA and ±30 mins outdoor time. 

**Table 1:** Descriptive statistics for participants according to the two independent variables tested in the main effect analyses.

	<30 min PA	30+ min PA	<30 min outdoors	30+ min outdoors
N	68	49	53	64
% men	27.0	36.0	30.2	29.2
	Mean (SD)	Mean (SD)	Mean (SD)	Mean (SD)
Age	85.1 (6.7)	80.6 (6.1)	83.4 (6.4)	83.0 (7.2)
PA time (min)	14.2 (7.8)	54.4 (24.1)	24.4 (19.6)	36.8 (29.3)
Outdoor time (min)	89.4 (145.2)	78.6 (109.2)	11.4 (9.0)	144.6 (152.0)
CESD score	6.5 (4.3)	3.9 (3.3)	6.6 (4.1)	4.4 (3.7)
PQoL score	3.8 (0.7)	4.1 (0.6)	3.8 (0.7)	4.1 (0.6)
FES-I score	2.2 (0.8)	1.5 (0.6)	2.2 (0.9)	1.8 (0.7)
Trails B time (s)	170.2 (7.8)	119.2 (57.8)	159.0 (77.6)	138.9 (75.5)
400MWT (s)	450.0 (199.9)	374.3 (95.0)	437.4 (209.6)	393.4 (152.0)
LLFDI score	30.3 (8.4)	39.1 (8.0)	31.3 (9.6)	36.8 (8.2)

The standard deviations for the time spent in PA or outdoors support the decision to create the categorical variables for analyses. Demographics were similar across those who spent ±30 min outdoors per day. Younger participants and men were more likely to be active 30 or more minutes per day.

In summary, we tested the main and interaction effects of the two dichotomous independent variables, PA and outdoor time, adjusting for covariates. PA and outdoor time were significantly related to depression, fear of falling, and self-reported physical functioning. PA alone was significantly related to 400 m walk time. All other results were not significant at *p* < 0.05.

Main effects were found for several of the outcomes. The depressive symptom scores were significantly different by outdoor time (F = 2.9; *p* = 0.012) and PA time (F = 4.7; *p* = 0.03). Participants who spent 30 or more minutes outdoors were more likely to have fewer depressive symptoms as did those who spent 30 or more minutes in PA. QoL scores were not significantly different across time spent outdoors or time in PA, but there was a trend in the hypothesized direction. Those who spent 30 or more minutes outdoors (F = 3.3; *p* = 0.07) and 30 min in PA (F = 3.5; *p* = 0.06) were more likely to report higher quality of life scores. Fear of falling was significantly different across both outdoor time (F = 5.6; *p* = 0.019) and PA (F = 9.9; *p* = 0.002). Those who spent 30 min outdoors or in PA reported less fear of falling. TMT-B scores were not significantly different for outdoor time or PA, although a trend was seen for the latter (F = 3.2; *p* = 0.08). Time to complete the 400m walk was significantly different by PA time (F = 5.9; *p* = 0.017) with a trend for outdoor time (F = 2.8; *p* = 0.09). Participants who spent more than 30 min being physically active completed the 400 m walk more quickly. The LLFDI score was significantly different across both outdoor time (F = 7.6; *p* = 0.007) and PA (F = 10.4; *p* = 0.002). Those who spent more than 30 min outdoors or in PA reported higher functioning scores.

**Figure 1:**
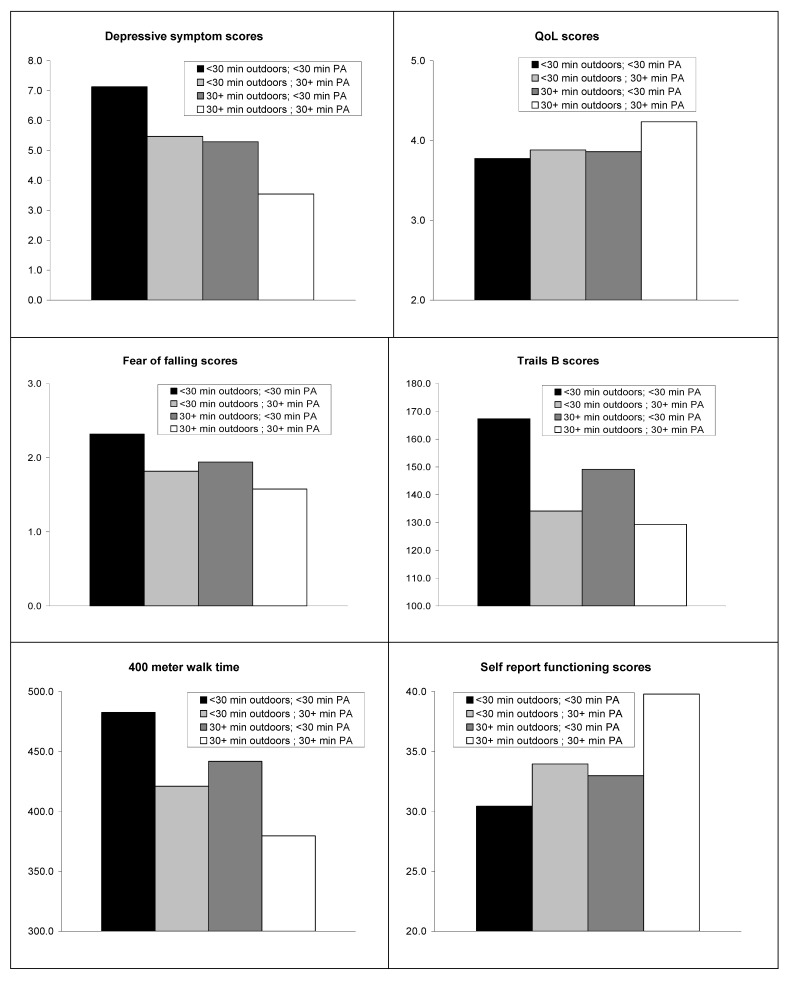
Mean scores for the four groups tested in the PA by outdoor time interaction.

Figure 1 presents the mean scores for the four groups tested in the interaction: (1) outdoor mins < 30, PA < 30 min; (2) outdoor min < 30; PA min 30+; (3) outdoor min 30+, PA min < 30; (4) outdoor min 30+, PA min 30+. The interactions of outdoor time by PA time were not significant for any outcome, thus any associations could be the result of random chance. However, the consistent pattern of the means across all the outcomes is highly informative given these are exploratory analyses and the combination of GPS and accelerometer data is being presented for the first time in relation to health indicators. Of particular interest is the pattern that those who were **not** active for at least 30 min and did **not** spend 30 min outdoors reported the highest levels of depressive symptoms (F = 0.006; *p* = 0.98), lowest scores for PQoL (F = 1.3; *p* = 0.3), highest times to complete the TMT-B (F = 0.12; *p* = 0.73) and 400MWT (F = 0.000; *p* = 0.99), had the greatest fear of falling (F = 0.23; *p* = 0.64), and lowest self-reported physical function (F = 1.3; *p* = 0.26).

## 4. Discussion

The study is one of the first to report the use of accelerometer and GPS data to concurrently assess PA location in older adults [37]. The study demonstrates it is feasible to use these devices to objectively assess outdoor time and PA, even in an older old population (average age 83).

Being outdoors and being active were both related to greater self-reported physical functioning, less fear of falling and fewer depressive symptoms. The emotional functioning outcomes replicate previous studies that have employed self-report measures of PA and outdoor time [6]. Studies have also shown that going outdoors can have long term functional health benefits for older adults [38,39]. The relationship between Vitamin D and bone health is well established [40].

One study that employed accelerometers and self-reported activity location found that older adults who exercise outdoors exercised for longer [41]. Exact mechanisms remain unknown but evidence suggests that greater enjoyment and opportunities for more social interaction may contribute to the outdoor activity experience [42]. One study in a large US national sample found that leisure time PA was related to higher Vitamin D levels in older adults [43]. Vitamin D deficiency is related to many chronic conditions including cancer, heart disease and bone health [36]. If older adults spent more time outdoors they may be more active and benefit from a healthy dose of Vitamin D.

These cross sectional data do not shed light on the direction of the relationship, people who are more confident and have higher functioning may be more likely to go outdoors or be active, as well as those who are active or go outdoors may be more confident and have greater functioning. The current intervention study, from which baseline data were employed, will be able to shed light on the causal relationship in future analyses when follow up data are available.

There was no statistically significant benefit of being outdoors and being active on the selected health indicators. However, the figures demonstrate that those who were both active and spent time outdoors presented with better outcomes. We found that people who spent more time outdoors were more likely to active. However, in this sample less than a quarter of the activity was performed outdoors so future analyses could look specifically at outdoor PA. Since so little activity was performed outdoors, the opportunity exists to intervene on this behavior and encourage more outdoor activity. Currently, limitations in physical functioning, fear of falling and neighborhood design may prevent older adults from being active outdoors so safe environments to support such activity will be crucial [44,32].Study limitations include the small sample size and homeogenity of this well-educated sample. It is possible that some outdoor activities may expose older adults to pollutants that can adversely affect health; this was not measured in the current study. The causal nature of the relationship also cannot be tested in this cross sectional data. This study confirms previous data that PA, beneficial for health and that outdoor time is related to emotional and physical functioning. This study employed new techniques to objectively assess outdoor PA, and demonstrates the utility of such measures. A study of outdoor *versus* indoor PA would provide clearer evidence of a synergistic effect of outdoor PA.
